# Clustering approach for the analysis of the fluorescent bioaerosol collected by an automatic detector

**DOI:** 10.1371/journal.pone.0247284

**Published:** 2021-03-11

**Authors:** Gintautas Daunys, Laura Šukienė, Lukas Vaitkevičius, Gediminas Valiulis, Mikhail Sofiev, Ingrida Šaulienė

**Affiliations:** 1 Šiauliai Academy, Vilnius University, Šiauliai, Lithuania; 2 Finish Meteorological Institute, Helsinki, Finland; Institute for Biological Research, SERBIA

## Abstract

Automatically operating particle detection devices generate valuable data, but their use in routine aerobiology needs to be harmonized. The growing network of researchers using automatic pollen detectors has the challenge to develop new data processing systems, best suited for identification of pollen or spore from bioaerosol data obtained near-real-time. It is challenging to recognise all the particles in the atmospheric bioaerosol due to their diversity. In this study, we aimed to find the natural groupings of pollen data by using cluster analysis, with the intent to use these groupings for further interpretation of real-time bioaerosol measurements. The scattering and fluorescence data belonging to 29 types of pollen and spores were first acquired in the laboratory using Rapid-E automatic particle detector. Neural networks were used for primary data processing, and the resulting feature vectors were clustered for scattering and fluorescence modality. Scattering clusters results showed that pollen of the same plant taxa associates with the different clusters corresponding to particle shape and size properties. According to fluorescence clusters, pollen grouping highlighted the possibility to differentiate *Dactylis* and *Secale* genera in the Poaceae family. Fluorescent clusters played a more important role than scattering for separating unidentified fluorescent particles from tested pollen. The proposed clustering method aids in reducing the number of false-positive errors.

## Introduction

Airborne pollen dispersal has been assessed using different technologies and methods [1–4]. Morphological properties allow the identification of airborne pollen by manual microscopy [2] or from the images and laser-induced fluorescence data collected by an automatic detector [4–6]. The overview of the pollen monitoring stations published by Buters et al. [7] showed that aerobiological observations had been made in almost nine hundred sites worldwide. About two-thirds of them had used the standardized Hirst-type method [8]. Using this method, pollen concentrations are obtained once a week, though shorter periods are possible. The shortcoming of this method is that sample preparation and identification require human resources proportionate to the sampling frequency. The growing demand for personalised information, forces to find new ways to obtain aerobiological data faster [9].

In recent years, there is increasing attention towards using automatic particle detectors to measure the dispersal of pollen in the air. The near real-time airborne pollen monitoring has been tested by scientists from different countries [4, 10–13]. While research results demonstrate a positive perspective of the ground-breaking technology, challenges in classifying particles of biological origin are often encountered. Kawashima et al. [10] show an algorithm for counting several kinds of airborne pollen grains simultaneously and noted that any particle with sideways and forwards scattering similar to those of the target pollen is misrecognized as target pollen. Pollen features is an unsolved question in calibration experiments, as fresh and dried pollen can generate different signals [4].

The growing network of researchers using automatic pollen detectors has the challenge to develop new data processing systems, making maximum application of pollen or spore data obtained in near-real-time. The particle size and shape are important morphological parameters describing pollen and are the relevant assessment units in most airborne particle detectors. Pollen is identified from particle images, made by different automatic devices [4, 5] according to its characteristic morphological parameters. The devices that collect data in real-time and operate using laser-induced fluorescence ensure a specific particle’s recognition. In some cases, particle identification is less accurate when an algorithm based only on particle fluorescence or scaterring is used. All these features are helpful for identifying particles and the combination of characteristics leads to the best results. The previous study demonstrated [13] that methods using fluorescence and scattering data performed better than methods using each measuring modality separately. Huffman et al. [14] note that the fluorescence spectra of individual molecules in the biological aerosol are broad and depend on many molecules, which complicates species-level identification from the fluorescence spectrum alone. Scattering images can be important for classification of pollen, especially distinguishing them from other fluorescent airborne particles of biological or non-biological origin. However, it remains relevant to appropriately choose data analysis methods that would improve or make better use of identification possibilities provided by automatic particle detectors, especially for identifying pollen or spores in the air.

Cluster analysis, as a method, has been successfully applied in large datasets and allows to group data according to emerging common characteristics [15] and can therefore be a convenient tool for evaluating scattered data that is difficult to group. In aerobiology, this method is used to assess airborne particles of biological origin while highlighting their dispersion patterns [16]. On the other hand, some clustering methods are applied not for identification of particles themselves but grouping particles of biological origin (e.g., pollen) according to concentration measurements in the air [17, 18] or for finding links with factors determining particle dispersal, such as weather conditions or long-range transport [16, 19–21]. Clustering also becomes a convenient tool for analysing the possibilities of automatic monitoring and assessing aerobiological station arrangement [12, 22]. Clustering particles of biological origin to analyse spectral data of single particles from a small test set of pollen types has a practical aspect, especially when seeking to improve the characteristics of fluorescence spectrometer sensors [23].

Automatically operating particle detection instruments generate many valuable data, but their use in routine aerobiology needs to be harmonized. Huffman et al. [14] analysed key challenges emerging at the intersection of the demand and possibilities of real-time technological advancement. Groups of researchers who have started using automatic particle detectors improve particle recognition algorithms, verify data [5, 6, 10], analyse the possibilities of their use in air quality models [24], and form new experience step by step. Some automatic particle detectors network development activities are being coordinated by the EUMETNET AutoPollen programme [25].

It is not always possible to recognise all particles constituting the atmospheric bioaerosol because of particles diversity. Previous research [13] implemented a neural network classification method, which was forced to assign every particle to a predetermined class. In this study, we aimed to find the natural groupings of pollen data by using cluster analysis, with the intent to use these groupings for further interpretation of real-time bioaerosol measurements. The results provide preconditions for more efficient identification of pollen from real-time data obtained by automatic particle detectors.

## Material and methods

### Environment of bioaerosol detection site

The bioaerosol studies conducted in 2019 took place in Šiauliai (55°55’34.4"N, 23°18’34.8"E), which is the fourth largest city (its territory covers an area of 81,13 sq. km, there are 100 thousand permanent residents) in Lithuania [26]. It is in the northern part of the East Samogitian plateau, about 140 km away from the Baltic Sea and the same distance from the Gulf of Riga. The green space system of the city of Šiauliai occupies 18,5% of the city’s territory, while free and undeveloped territories make up about 15% [27]. According to the municipal atmospheric monitoring data, the average concentration of gaseous pollutants and particulate matter in Šiauliai city in 2019 did not exceed the maximum levels [28]. During the pollen monitoring period, the highest PM_10_ concentrations per day were recorded in April and May.

### Particle data

In this study, two types of data were used: known pollen morphotypes tested in the lab (test data) and atmospheric bioaerosol observations in Šiauliai (observational data) within the February-October 2019 time frame. The same Rapid-E detector was used to collect all particle data. For each particle, the instrument records its scattered light pattern, 320 nm laser-induced fluorescence spectra, and fluorescence lifetime [29]. The scattering image is obtained by illuminating the particle with a 400 nm laser beam. For this study, we use fluorescing particles if they reach at least 1500 intensity at maximum fluorescence as registered by the device [13]. Laboratory tests were performed with woody (20 taxa) and herbaceous plant pollen (9 taxa) (Table 1).

**Table 1:** The list of plants whose pollen were tested under laboratory conditions.

	Family	Species
Woody plants	Aceraceae	*Acer negundo*
		*Acer pseudoplatanus*
	Betulaceae	*Alnus glutinosa*
		*Alnus incana*
		*Betula pendula*
		*Betula pubescens*
		*Corylus avellana*
	Cupressaceae	*Juniperus communis*
		*Juniperus squamata*
	Pinaceae	*Picea abies*
		*Pinus mugo*
		*Pinus sylvestris*
	Salicaceae	*Populus canescens*
		*Populus tremula*
		*Salix alba*
		*Salix caprea*
		*Salix fragilis*
	Oleaceae	*Fraxinus excelsior*
	Juglandaceae	*Juglans regia*
	Fagaceae	*Quercus robur*
Herbaceous plants	Asteraceae	*Ambrosia artemisiffolia*
		*Artemisia vulgaris*
	Poaceae	*Dactylis glomerata*
		*Festuca pratensis*
		*Festuca rubra*
		*Secale cereale*
	Plantaginaceae	*Plantago lanceolata*
		*Plantago media*
	Urticaceae	*Urtica dioica*

The plant taxa presented in the table are the most common airborne pollen types in Lithuania [30]. For tests inflorescences were collected in nature during the plant flowering period and gently dried in the laboratory for three days at 30°C. The procedure of pollen isolation from inflorescences was performed according to Šaulienė et al. [13]. Until the start of the experiment clean, impurity-free pollen was stored refrigerated at a temperature of 5 ± 1°C and relative humidity of 57 ± 10%. Test data is published at Zenodo [31, 32]. Aspergillus spore test data was also included in this study. Labels were assigned to test data according to its taxa.

Bioaerosol observational data (*in situ* data) was recorded in Šiauliai at 20 m above the ground, implementing aerobiological stations’ requirements [2]. Data obtained by conventional aerobiological methods [8] were used to select time windows with negligible pollen quantity or periods without pollen. Such time windows were selected from the periods when pollen was not detected by Hirst method. Fluorescent particle events were filtered from observational data within these time windows. Approximately 1.5 million fluorescent particle events gathered this way were assigned as an unidentified fluorescent particle (UFP).

### Principles of clustering

A clustering approach was chosen to investigate the similarity between particles observed by Rapid-E device. We aimed to find natural groupings therefore customary clustering methods (as K-means), which need to know the number of clusters in advance, were not suitable for the task. As a result, we selected agglomerative clustering from the hierarchical clustering class [33]. Agglomerative clustering finds a number of clusters itself by a given distance threshold value.

**Fig 1:**
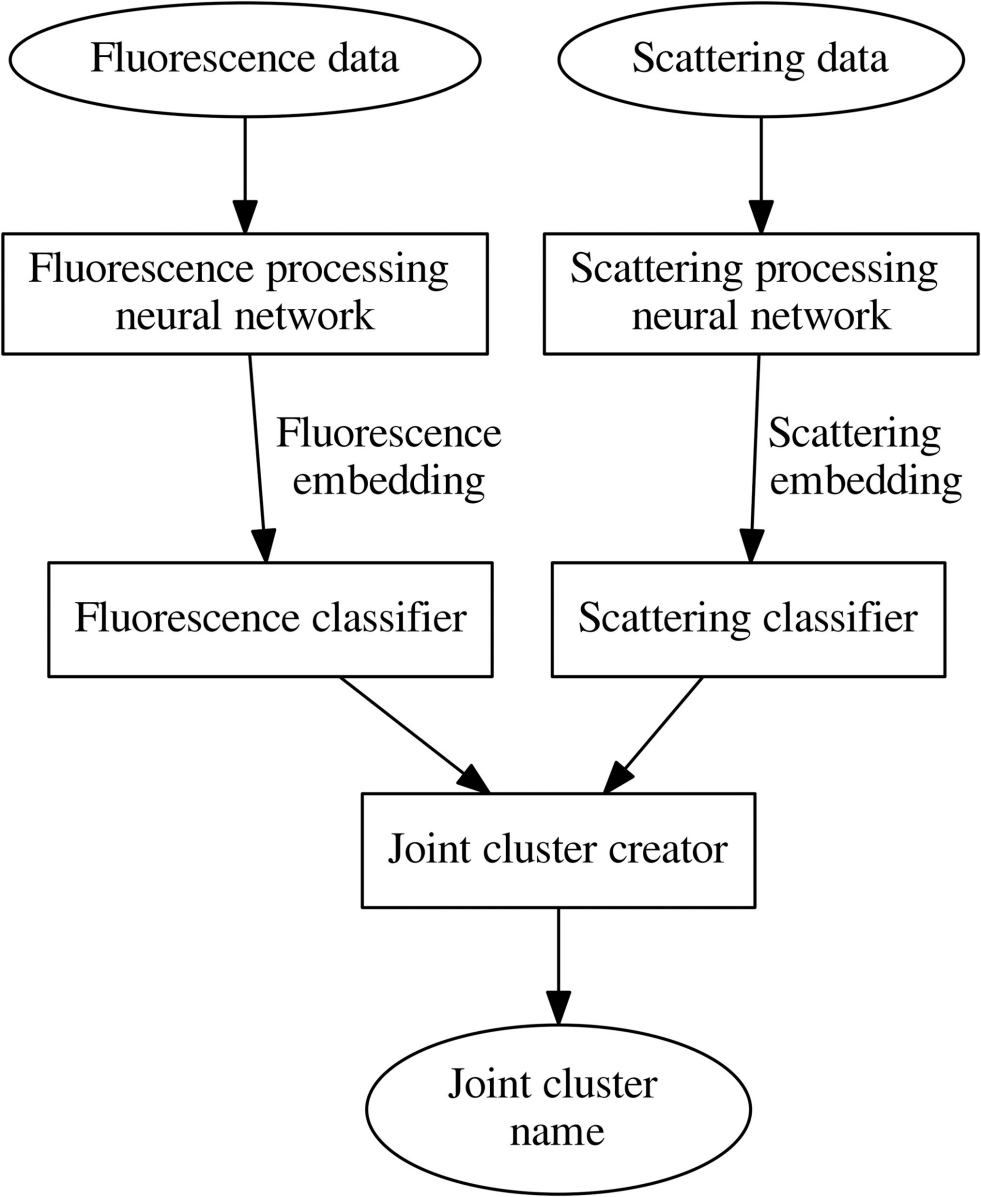
The principal clustering scheme.

The principal clustering scheme is shown in Fig 1. Because recent research [13] revealed that neural network classification approach is promising for recognition of pollen taxa registered by Rapid-E device, we wanted to preserve advantages of this approach. Therefore, as clustering features, we selected fixed length continuous number vectors—embeddings (following notation of deep learning) obtained using two neural networks. One network produces embedding from the particle fluorescence spectrum; the other, from scattering image data.

Detected embeddings are forwarded to classifiers that identify fluorescence and scattering clusters. Classifiers calculate the cosine similarity with the classifier’s cluster centres and select the cluster with which the cosine similarity of embedding is the greatest. Finally, the name of the joint cluster is identified. The name of the cluster consists of a Latin letter that indicates the name of the scattering cluster and a number that indicates the number of the fluorescence cluster. The block joint cluster creator verifies whether the cosine similarities satisfy the threshold value and derives the name of the joint cluster to which the particle is assigned.

Neural network architectures and training process is described in work [13]. A multilayer perceptron was used for raw fluorescence data processing. A neural network for scattering data processing has a sequence of two convolutional blocks for the feature extraction and fully connected layers for extraction of embeddings. During training, multilayer perceptron had 3 fully connected layers, scattering processing neural network—2 fully connected layers. The last layers of every neural network are not used during embedding extraction. Instead, a normalization layer is added to every network to produce unit length embeddings.

Next step to implement the proposed algorithm is to obtain cluster canters in the embeddings space. Agglomerative clustering applying the cosine similarity metrics was performed on embeddings extracted from testing data. Trial and error method was used to find a threshold for clustering. The objective was to avoid splitting of a group of the same pollen taxa to too many clusters. Empirically for our purpose, the most suitable threshold was 0.6. Certainly, the threshold is sensitive to the way embeddings are processed, and a change of neural network architecture could invoke the shift of threshold. After clustering, centres of the cluster were found as mean values of cluster embeddings.

As a result, we found that scattering data could be grouped into 8 clusters, which we have given letter names (A-H). Fluorescence embeddings could be grouped into 16 clusters, which were named after cluster numbers. The joint cluster name is obtained by adding the fluorescence cluster number to the scattering cluster name (e.g., E5). We obtained a total of 128 joint clusters. Clustering was implemented using the Python programming language in the Anaconda environment. The PyTorch framework was used for the development and training of neural networks. The Scikit-learn module was used for agglomerative clustering. Each of the tested (Table 1) pollen taxon data fell into various joint clusters. The obtained cluster results are arranged according to the number of particles grouped in them. The joint clusters with the highest number of the tested pollen were selected as dominant. Pollen clustering was performed without attention to seasonal pollen load or prior knowledge about the regular pollen seasons of each taxon. All the clusters were developed by using data from pollen experiments in the laboratory. Bioaerosol observation data were included to associate airborne particle data (recorded in by Rapid-E real-time) with distinguished clusters.

The results of the experiments conducted in the laboratory, grouped into clusters, were visualized using R [34] and R Studio [35] software along with ‘ggplot2’ [36], ‘ggrepel’ [37] packages. In the analysis of the results, the difference between the dominants of clusters is expressed in percentages. To demonstrate the clustering value in the real-time data analysis, mainly joint clusters of the most important pollen taxa were associated with data registered by the automatic particle detector.

## Results

### Clustering of tested fluorescent particles

**Fig 2:**
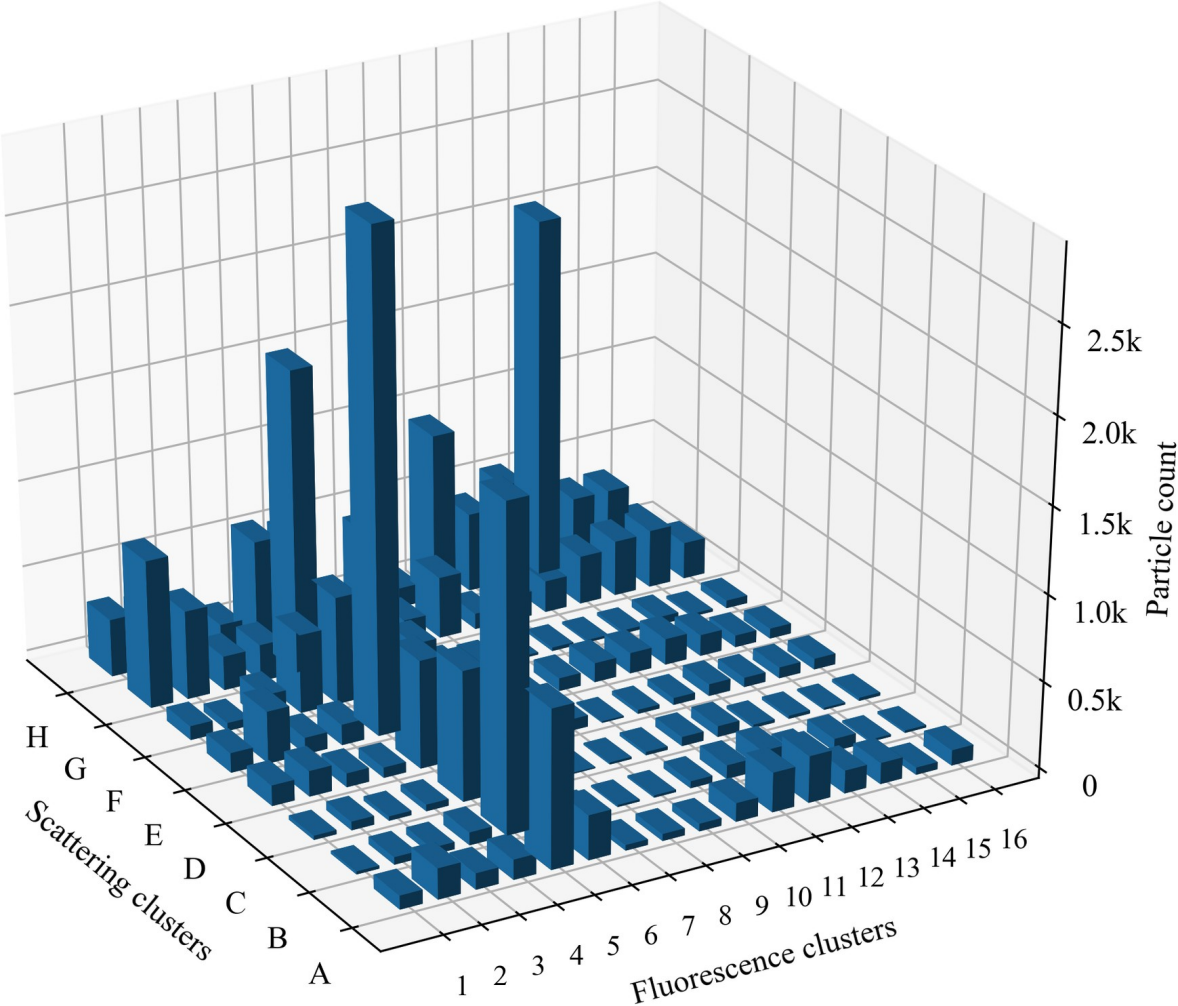
Distribution of fluorescence clusters and scattering clusters of pollen tested under laboratory conditions. Each of the pollen taxa is represented by 1000 particles.

The results of the clustering of pollen testing data performed under laboratory conditions are presented in Fig 2.

**Fig 3:**
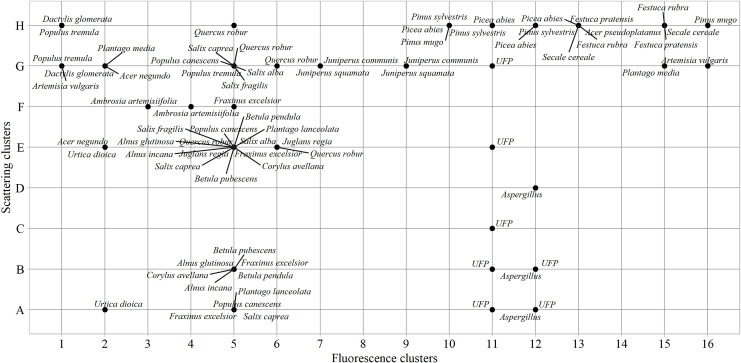
Clusters of pollen and spores tested under laboratory conditions. Unidentified fluorescence particles (UFP) displayed as additional information formed from ambient bioaerosol observational data.

In the pollen library [31, 32], we randomly selected the same number of pollen– 1000 particles, clustered according to scattering and fluorescence properties, for each tested plant species (Table 1). The Fig 2 illustrates the clusters that distinguish themselves by the abundance of tested pollen, in which pollen is grouped according to the properties characteristic to a particular cluster. Fluorescence results reveal 3 clusters containing the bulk of the total particles: 5, 11, and 13. The result of the tested pollen allows for the application of cluster analysis. One of the cluster results (scattering or fluorescence) obtained with the particle detector is less sufficient–it is necessary to combine them into joint clusters. This assumption is reinforced by fragmentation in cluster results of tested plant species pollen (Fig 3). For example, if clustering based only on scattering data, *Urtica dioica* pollen could be grouped with *Aspergillus* spores in the same cluster (A cluster). If scattering clusters are combined with fluorescence clusters, two specific joint clusters A2—*Urtica dioica*, A12—*Aspergillus* composed. If using only fluorescence clusters, *Urtica dioica* and *Plantago media* pollen cannot be distinguished, because both are in cluster 2. Scattering clusters allowed grouping pollen in specific joint clusters (G2—*Plantago media*, A2—*Urtica dioica*).

Fluorescent airborne particles that were not assigned to any of the tested pollen taxa (presented in Table 1) or *Aspergillus* spores were grouped as “unidentified fluorescent particles”. Of 29 analysed taxa, the largest diversity of pollen types is concentrated in the fifth fluorescence cluster. All tested species belonging to the Betulaceae and Salicaceae families fall into this cluster but are ambiguously distributed according to scattering results. Pollen from eight different taxa falls into the joint cluster E5, which is significant but not always the main cluster; i.e., the one with the maximum amount of tested pollen. Most of the *Corylus*, *Alnus*, and *Betula* pollen data are in joint clusters B5 and E5. Analysing Betulaceae pollen data registered by the automatic detector, the joint cluster E5 contained up to 25% more particles than B5. Pollen of the *Populus* and *Salix* was more than twice often associated with the cluster G5 than with the cluster E5. This suggests that while grouping pollen data, the joint cluster G5 can be used as a marker for the Salicaceae family. Another specificity was found regarding *Fraxinus excelsior* present in the fifth fluorescence cluster. The results of pollen, tested in the laboratory, showed that five of eight scattering clusters were related to taxon identification: mostly grouping takes place in the joint cluster E5, but slightly fewer results concentrate in scattering clusters A-C and F.

Unlike broadleaf plant pollen, conifer pollen data grouped into other clusters that are specific to them. For example, twice more particles of *Juniperus* grouped in the joint cluster G7 than in G9, which becomes the second cluster characteristic to pollen in this genus according to the abundance of particles. It should be noted that the results of coniferous plant pollen remain in the same scattering cluster but are distributed among different fluorescence clusters. The basis for this statement is the case of *Juniperus* and the data of Pinaceae pollen. Fig 2 shows that the pollen of this taxon is in the scattering cluster H, but *Pinus* falls into 10–12; while *Picea*, into fluorescence clusters 10–13.

Similarly, as with pollen of woody plants, herbaceous plant pollen quite clearly groups concerning the family taxon. Taking note of the joint clusters of pollen from the Asteraceae and Poaceae plant families, the distribution into scattering clusters G and H, respectively, emerges, which can be considered a marker. Besides, available results demonstrate that the *Dactylis glomerata* fluorescence cluster differs from *Festuca*, which potentially allows classifying pollen from Poaceae family plants by genera.

Results presented in Fig 3, shows clustering according to the size and shape of the tested pollen. Of the tested 29 pollen and spore taxa, three groups are formed, covering small (> 10 μm), medium, and large (> 50 μm) particles. The same taxon falls into several scattering clusters due to differences between diameter in an equatorial or polar view of pollen. For example, some of the smaller *Fraxinus excelsior* pollen in cluster A5 was most likely scanned in polar view, while the larger ones (clusters E5 and F5) examined at the equatorial oval view. The occurrence of *Populus canescens* pollen in the cluster A5 may be based on dehydration reason. Populus pollen is usually 25–40 μm [38], and dehydrated, deformed pollen is likely to be detected in the scattering cluster (A) of the small particles. Dehydration reason is likely related to the results of *Urtica dioica* when pollen from the same sample was divided into two clusters according to particle size: small (A2) and medium (E2) particles. Pollen from the Poaceae and Pinaceae families figured out by its size and tested pollen is in a scattering cluster H, which includes the largest pollen. In nature, Piceae pollen is twice as large as Poaceae. Our results show them a similar size because dry pollen was used in the laboratory experiments.

Pollen of similar size but different taxa can be more precise classified by fluorescence. For example, *Festuca pratensis* (H13) and *Secale cereale* (H13) pollen can be distinct from *Dactylis glomerata* (H1) pollen, although all of them belong to the Poaceae family and are difficult to separate by microscope. The fluorescence cluster also distinguishes large pollen of *Quercus robur* (H5) from the Pinaceae (H10-H12) pollen. The performed sorting shows that spores characteristically separate. In this study, only *Aspergillus* was tested; it falls into joint clusters A12, B12, D12. It is challenging to make premises about spores based on the data of one taxon, but the information is valuable for the development of spore recognition algorithms.

Fig 3 also included unidentified fluorescent particle clusters extracted from the observation data of 2019 by filtering as described in the material and methods section. This group clearly stands out from the pollen data stream, encompassing fluorescent particles that are not assigned to the tested pollen or spores. The data of unidentified fluorescent particles separate into the fluorescence cluster 11 and together with the scattering results form seven specific joint clusters. In Fig 2, these are A11-12, B11-12, C11, E11, G11.

### Clustering of bioaerosol recorded by the automatic particle detector in real-time

**Fig 4:**
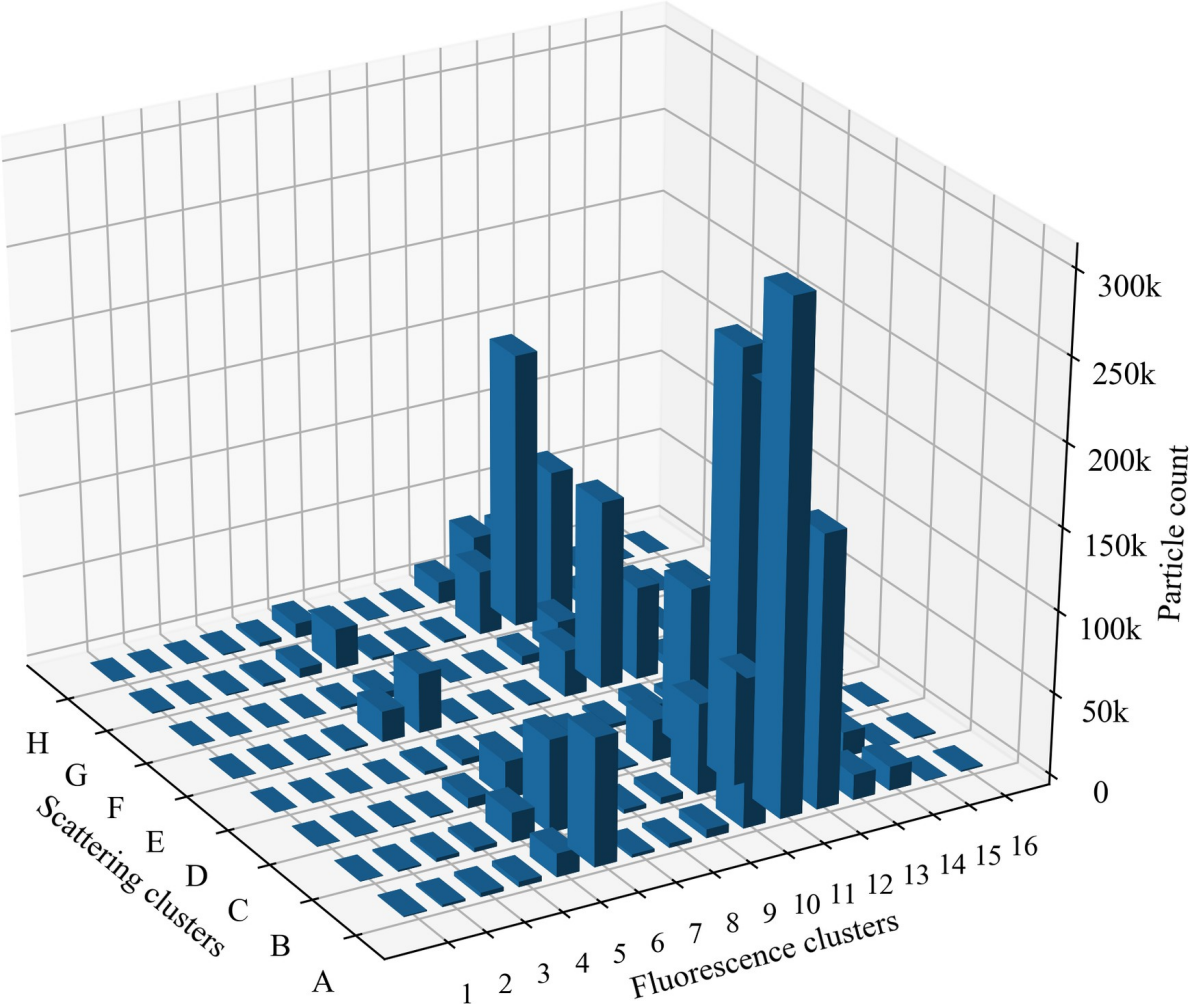
Distribution of joint clusters of fluorescent particles detected by the automatic particle detector in 2019. Letters mark scattering clusters, numbers indicate fluorescence clusters.

In 2019, the automatic detector Rapid-E operated in Šiauliai and recorded airborne particle data throughout the vegetation period (February-October). The second part of the study was conducted based on this data, which combined the principles of clustering of tested particles and enabled to evaluate clustering possibilities, using *in situ* real-time observation data. Thus, the data of fluorescent particles detected by the automatic particle detector in Šiauliai in 2019 were grouped into clusters (Fig 4).

Most of the particles detected in the atmospheric bioaerosol fall into fluorescence clusters 11 and 12, among which more cases belong to scattering clusters A-C. Based on testing performed in the laboratory (Fig 3), these scattering clusters do not contain significant data in defining pollen taxa. It is worth considering joint clusters H11 and H12 can be associated with pollen of the Pinaceae family. Throughout 2019, in the fifth fluorescence cluster related to a higher diversity of pollen taxa (Fig 3), fewer particles were recorded than in the above-mentioned clusters.

**Fig 5:**
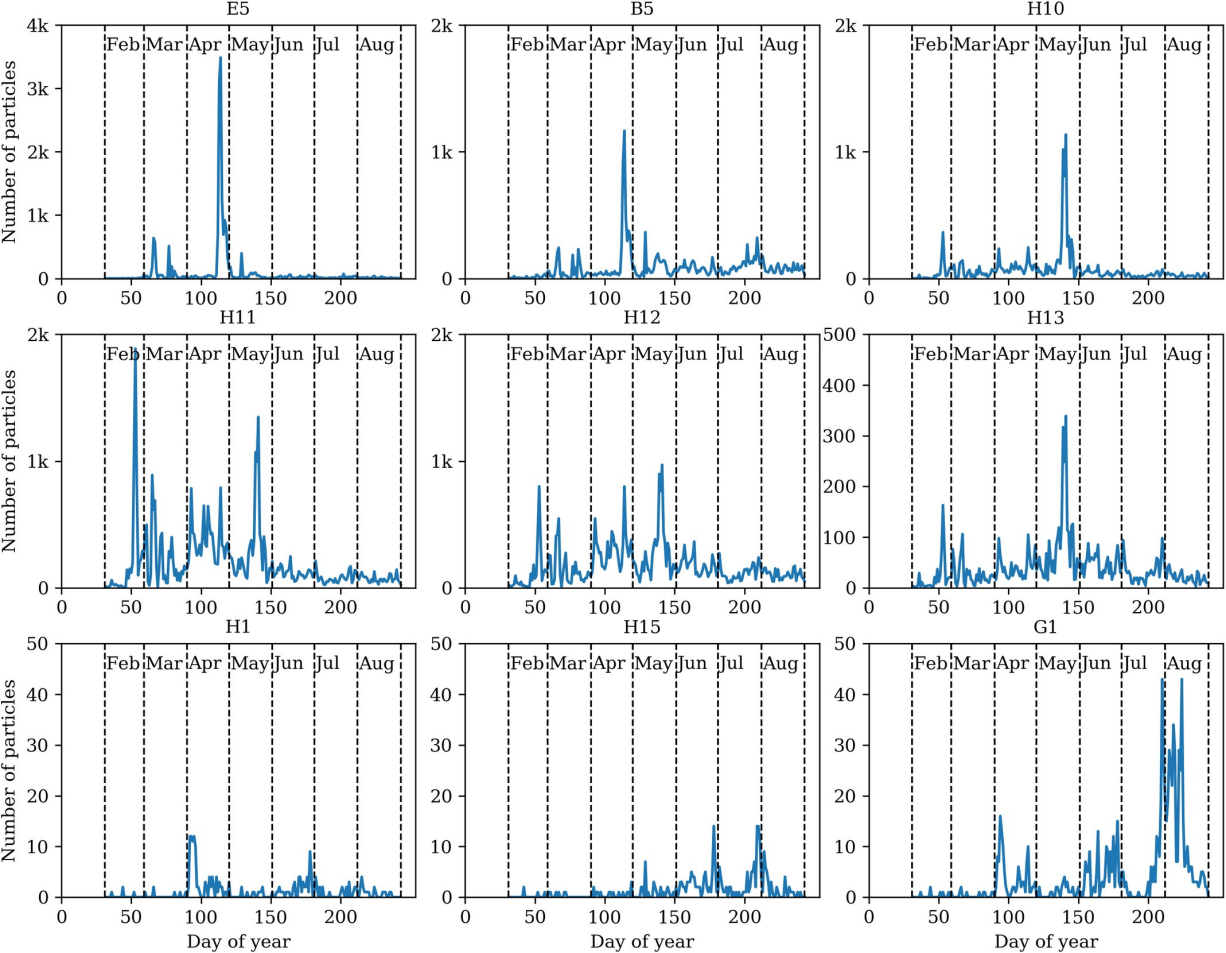
Dynamics of fluorescent particle clusters of bioaerosol observation data collected in 2019.

**Fig 6:**
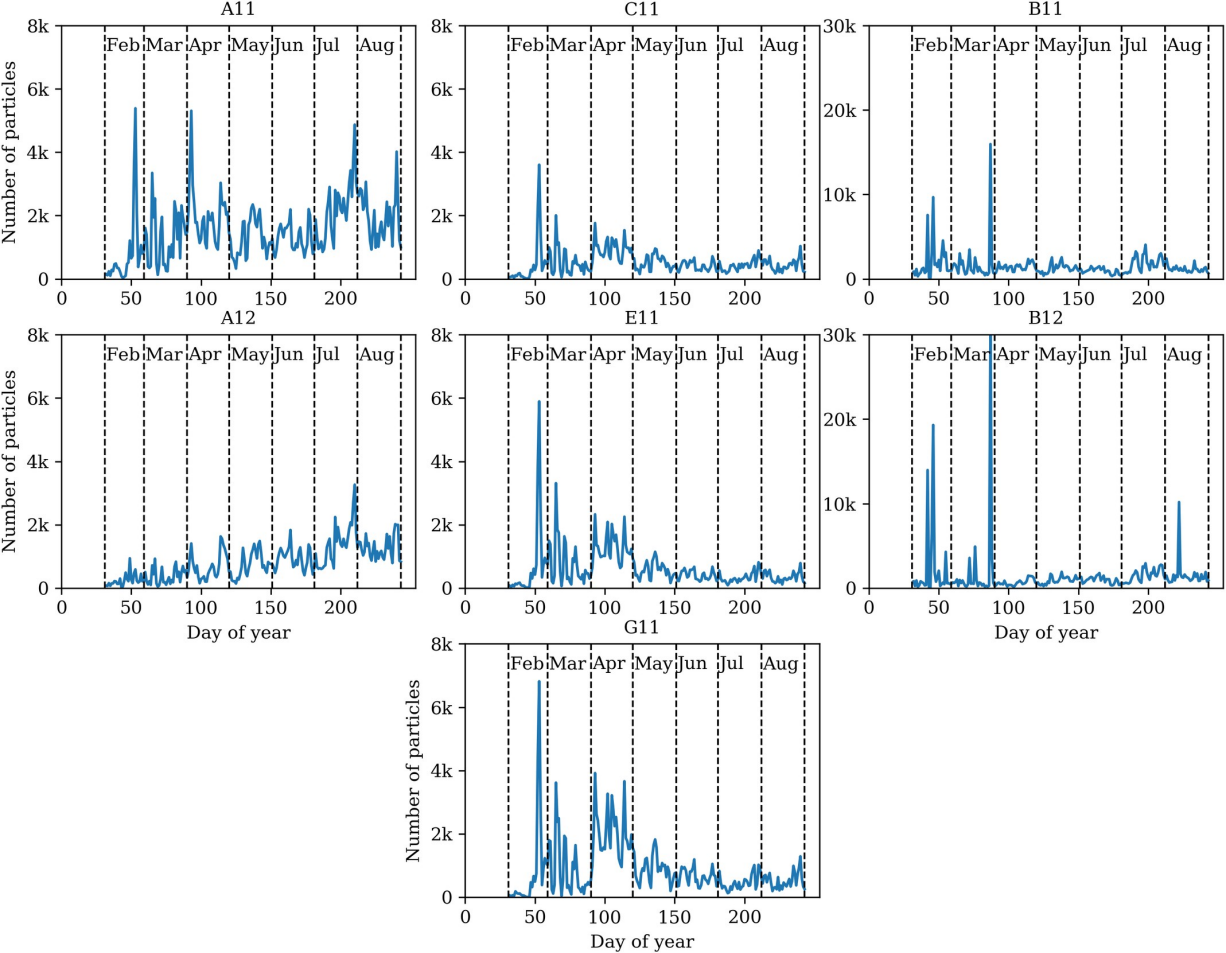
Dynamics of Unidentified Fluorescent Particles (UFP) clusters according to bioaerosol observation data collected in 2019.

To analyze the observation data (Figs 5 and 6), we selected the clusters containing the most pollen taxa (Fig 3). By associating the particles detected in the *in situ* observation with the clusters distinguished in the laboratory, we obtain indicative results in joint clusters E5 and B5. Experimental test data demonstrate that pollen of *Alnus*, *Betula*, *Fraxinus*, *Populus*, *Quercus*, *Salix* fall into joint cluster E5. The observed fluorescent particles annual curve (Fig 5) of E5 cluster mirrors the flowering time [39] of the named plants.

In the second part of the plant vegetation season (from day 150 of the year in Fig 5 E5), small amounts of particles defined by this cluster can be partially associated with pollen scattered by *Plantago* belonging to the cluster E5. The results of the cluster B5 are quite equivalent as well: the day with the highest number–more than 1000 –of particles corresponds to the period of flowering of birches [40, 41], but the second half of the year is less characteristic, compared to the data of tested pollen. This result could be possibly revised by a greater variety of tested pollen from herbaceous plants in the future. The H10 cluster is indicative concerning *Pinus* pollen quantity (Fig 3). This is displayed by the dynamics of the data recorded in real-time, i.e., pollen amount in the cluster fits the pine pollen season [42–44]. Coniferous plant pollen also fell into clusters H11-H13 in testing experiments. The annual dynamics of the cluster H13 can be treated as relatively more expressive (the dominant particle peak in May), but cases H11 and H12 are still difficult to interpret.

The last three graphs of the clustering of real-time observation data (Fig 5, H1, H15, and G1) clearly display lower fluorescent particles levels per day. According to laboratory tests (Fig 3), Poaceae pollen falls into these clusters and spreads less pollen than flowering trees. This is typically reflected in the data analysis of particles detected by Rapid-E in 2019. A higher number of fluorescent particles in the cluster H1 is possibly related to Populus pollen. In the second half of the plant vegetation season (from day 150 of the year), the rhythm of Poaceae flowering (pollen spreading) can be seen in cluster H1. June and July months (H15 cluster) results may be associated with the increase in pollen, characterised by meadows and crops (including *Secale*) flowering [39, 42]. The curve of the pollen concentration of *Artemisia* (plants start flowering in the second half of July) is reflected in the dynamics of particles assigned to the cluster G1.

In 2019 bioaerosol observations performed with the automatic particle detector showed that a large part of the airborne fluorescent particles detected, could not be associated with the tested pollen and spores (Table 1). UFP is in several clusters (Fig 6). Noteworthy to indicate, that these particles (Fig 3) slightly vary in terms of fluorescence but are quite widely dispersed in size. Analysing the distribution of UFPs over the year, several variations are established (Fig 6). One relates to clusters A11 and A12, where the number of particles had a dynamical grow from spring and reached its maximum in the second half of summer. UFPs of the same fluorescence but slightly higher in size (B scattering cluster) form the significant number of unidentified particles in this study. Fig 6 displayed that the dynamics of the particles in B11 and B12 clusters are particularly closely related to each other. Results allow presupposing that fluorescent particles could be of the same type but slightly different in particle size. It is even more complicated to explain the UFPs concentrated in the C, E, and G scattering clusters. Following the annual dynamics of particles, it is evident that the highest number of airborne particles was recorded in late winter. During April the increase of fluorescence particles in those three clusters could be mistakenly linked to the pollen that usually abundant in this month [39]. Combining scattering clusters with fluorescence clusters revealed that none of the tested pollen types falls in the E11, C11, G11 joint clusters (Fig 3).

## Discussion

This study has shown that pollen clustering is a suitable tool enabling a sufficiently correct grouping of airborne fluorescent particles data. Gained knowledge is important, seeking to harmonize the identification of allergenic pollen or fungal spores in real-time. Tešendić et al. [45] indicated that classifications of bioaerosol observation data have a notable amount of false-positive detections that was eliminated by manual limitation of the pollen season. Challenges arise from the specifics of the neural network as a "black box". The number of false-positive errors can be reduced if the clustering method applied after primary data processing by neural networks.

Airborne particles detected by automatic devices [4–6, 10, 46] are identified using different approaches and methodologies. Each device is tested with the set pollen or fungal spores selected for the specific study, and summarized results are published in scientific publications. Our openness strategy allows anyone to benefit from laboratory experiments data of tested pollen with the Rapid-E device [31, 32]. The developed digital data library is valuable material [13, 47] and allows successfully improve the knowledge on the features of particles present in the atmospheric bioaerosol. This study has revealed that the clustering of pollen data in the stage of identification is valuable in various aspects. One example is new knowledge of the different properties of conifers and broadleaf plants pollen. Pollen of broadleaf plants more often concentrates in the same fluorescence cluster. Conifer plant pollen was more similar in terms of scattering clusters but falls into different fluorescence clusters. Another example is related to Poaceae pollen features. The obvious specificity of grasses pollen suggests that the benefit of using automatic particle detectors may include not only real-time data flow but also more accurate grass pollen recognition. Our experimental data demonstrate the potential to group Poaceae pollen to the genus level (Fig 3). Performing the current study, we demonstrated that *Festuca* and *Dactylis* could be separated in real-time because fluorescence features are slightly different. So far, there have been few comprehensive studies analysing this possibility. Typically, 1–2 genera are selected from one plant family, or one species is selected as a representative. Pollen load information at the family level meets the needs of only a part of people with allergies [48]. O’Connor et al. [11], found differences in the fluorescence profile between *Agrostis stolonifera* and *Poa pratensis*. Results of this study provide promising results with Poaceae, but future validations are obligatory. Moreover, the more precise recognition with a clustering approach is demonstrated by identifying the species level *Pinus sylvestris* and *Pinus mugo*. This feature might have been related to the ability of Rapid-E to use fluorescence characteristics for particle detection. The chemical fingerprints of *Pinus* species that have morphologically undistinguishable pollen are clear, which makes it possible to differentiate the pollen of *P*. *sylvestris* and *P*. *mugo* [49]. However, researchers assessing the quality of atmospheric aerosol data observed by automatic detectors more often associate results concerning one species or genus [4–6], but there is a lack of specialized research.

Analysing the bioaerosol observation data, many fluorescent particles were grouped in unidentified fluorescent particles clusters. They differ in the abundance of particles that have fallen into each cluster. Based on the available data, it looks likely that pathogenic spores or airborne particles that have not been tested in the laboratory might have entered unidentified fluorescent particles clusters. Such an assumption is formed because *Aspergillus* forms a cluster resembling unidentified fluorescent particles (Fig 3). Even though pollen of 29 plant species was tested in the laboratory, the obtained background results of unidentified fluorescent particles require increasingly detailed experiments with pollen from anemophilous plants that have not been tested so far (e.g., *Quercus petraea*, *Solidago virgaurea*, *Chenopodium album*, and other) or with microscopic fungal spores (e.g., *Alternaria*, *Cladosporium*).

The use of clusters for the analysis of fluorescent aerosol has supplemented the results of our previous studies [13, 47] and provided new knowledge on the use of the automatic detector for operational information on airborne pollen concentration. Occasionally, authors [5, 6, 13] encounter unexplained cases due to specific identification issues related to pollen features. Cluster analysis has also demonstrated the necessity to study peculiarities of pollen properties in future. Based on the research results, pollen clustering could be used as a marker for identification of plant family or genus and could be helpful in the development or management of air quality models. This assumption is supported by analysing bioaerosol observation data performed with the automatic detector in 2019 and joint clusters results (Figs 5 and 6). In this study, clusters of pollen from the same taxa originated in different geographical areas is not compared but plan to do this in the future. We previously observed that the fluorescence properties of *Alnus* pollen change due to ozone exposure [47]. It is also known about Fagaceae high species‐specific differences in pollen’s chemical composition and high phenotypic plasticity due to either location or year [50]. There are still questions about the variation of fluorescence over time due to the possible changes in pollen fluorescence properties; therefore, clustering results for modelling purposes is still not straightforward.

In this study, we revealed that clustering is an appropriate complementary method to improve the interpretation of the results from neural networks. However, this is just one of the studies relevant to automated pollen identification, and it provides not only answers but also raises issues for future research. It is currently uncertain how to deal with pollen of different taxon grouped in the same joint clusters. Perhaps identification success rates will improve if more clusters are distinguished. It is equally important to conduct additional studies in which the clustering method is applied after primary data processing by neural networks to improve the identification of individual taxa from bioaerosol observation data that was collected by automatic detectors without external interpretation of pollen seasonality.

## Conclusions

Bioaerosol observational data collected by automatic particle detectors include a variety of airborne particles. Within the diversity of bioaerosol particles registered by device is a relatively low amount of pollen. Separation pollen from the fluorescing particles involves several stages of data processing. If the required quality of results is not reached with the clasiffication using neural networks, the clustering method could be used for fillering false positive events. Based on pollen and spore data obtained from laboratory tests, clustering neural network output data allowed to find the natural grouping of data, without additional constraints. The natural grouping of pollen by using agglomerative clustering allows the creation of fluorescence and scattering clusters. Scattering clusters are associated with particle size and shape properties, so pollen data are naturally grouped into several categories. Due to the peculiarities of the pollen shape in polar and equatorial view, pollen from the same taxon is assigned to different scattering clusters. We found that pollen from different taxa can fall in the same scattering cluster, possibly because of dehydration or particle shape. Betulaceae genus pollen data grouped in the same clusters based on fluorescence properties. Contrarily, the Poaceae family pollen fell in separate fluorescence clusters. The natural grouping of pollen demonstrated that it may be possible to differentiate *Dactylis* and *Secale*.

Analysing bioaerosol observation data, an important role of fluorescence has been established in distinguishing pollen data from other, unidentified fluorescent particles. Unidentified fluorescent particles fall in fluorescence clusters distinct from pollen and according to the results of scattering clusters are different sizes and shapes. Clustering data highlighted cases where identification is confusing and challenging to solve until more specific knowledge about the properties of pollen and fungal spores will be achieved.

The clustering strategy for airborne fluorescent particles, demonstrated in this research, can be used to improve the development of algorithms, enabling more accurate identification of pollen by the new generation particle detectors. The proposed clustering method aids in reducing the number of false-positive errors after primary data processing by neural networks. It would be beneficial to perform research on the applicability of clusters by analysing bioaerosol data collected by automatic detectors of other countries.
